# Assessing COVID-19-related anxiety and functional impairment amongst nurses in Malawi

**DOI:** 10.4102/phcfm.v13i1.2823

**Published:** 2021-06-30

**Authors:** Genesis Chorwe-Sungani

**Affiliations:** 1 School of Nursing, Kamuzu University of Health Sciences, Blantyre, Malawi

**Keywords:** anxiety, COVID-19, nurse, prevalence, functional impairment

## Abstract

**Background:**

Psychological well-being of nurses is crucial for them to effectively discharge their duties. However, coronavirus disease 2019 (COVID-19)-related anxiety can interfere with nurses’ performance and reduce their self-efficacy.

**Aim:**

The primary aim of this study was to assess COVID-19-related anxiety and functional impairment amongst nurses in Malawi. The secondary aim of the study was to determine reliability and validity of the Coronavirus Anxiety Scale.

**Setting:**

The study was conducted in Malawi.

**Methods:**

This was a cross-sectional study that collected quantitative data from 102 nurses in Malawi online. Data were analysed using descriptive statistics and receiver operating curve analysis.

**Results:**

This study found that 25.5% (26) of respondents had COVID-19-related anxiety and 48% (49) functional impairment. There were significant differences in the numbers of respondents who had functional impairment in relation to workplace (*Χ*^2^ = 8.7, *p* = 0.03), with many of those working in hospitals (58.6%, *n* = 34) having highest levels (mean = 20.6 ± 10.4). The Coronavirus Anxiety Scale proved to be an effective instrument (Sensitivity = 73.1%; Specificity = 60.5%; area under the curve = 0.73) for assessing COVID-19-related anxiety amongst nurses.

**Conclusion:**

It is necessary to screen nurses for COVID-19-related anxiety and functional impairment and provide them effective psychosocial interventions. Policymakers should place more emphasis on allocation of financial resources to mental health services and staff support programmes targeting nurses during pandemics. There is a need to conduct future research on mental health interventions that might be used to assist nurses with COVID-19-related anxiety and functional impairment.

## Introduction

The coronavirus disease 2019 (COVID-19) is fast spreading across the globe, causing an outbreak. Healthcare professionals from various disciplines and cadres are involved in the care of patients with COVID-19.^1^ The high prevalence of COVID-19, its novelty and rapid spread, and the associated morbidity and mortality rates have acutely overstretched the limits of healthcare systems worldwide.^2^ This is a situation in Malawi where healthcare services have been overwhelmed from the first time a case of COVID-19 was detected on 02 April 2020. Data from Public Health Institute of Malawi reported by media indicate that as of September 2020, more than 5500 people have tested positive for COVID-19 and the numbers keep on rising. In addition, more than 150 people including one health worker have died of the disease in Malawi. In 2020, a media report from the Ministry of Health indicated that there are 67 nurses who have tested positive for COVID-19 in Malawi. The upsurge of COVID-19 patients has caused an increase in care demands on nurses both in hospitals and community.^2^ Consequently, it has negatively affected some nurses psychologically.

It is documented that nurses are now afraid of going to work because they fear for their lives and family.^3^ They suffer from great amount of anxiety every time they go for work because they fear that they will get sick of COVID-19 and end up dying like their patients.^3^ The high infection and mortality rates related to COVID-19 cause extensive fear and anxiety.^4^ As such, identifying high-risk groups for psychological symptoms is as important as recognising the presence of these symptoms, as they will be the target populations for evaluation and perhaps treatment.^5^ Frontline healthcare workers are particularly vulnerable to mental health problems associated with COVID-19.^6^ A systematic review reported a pooled prevalence of COVID-19-related anxiety amongst health workers as 23.2%.^7^ In Nepal, nurses were found to be significantly more likely to experience anxiety symptoms related to COVID-19 than other health workers (adjusted odds ratio [AOR]: 2.33; 95% confidence interval [CI]: 1.21–4.47).^8^ Coronavirus disease 2019-related anxiety can interfere with performance of nurses in their duties and reduce their self-efficacy levels.^9^ Psychological well-being of nurses is crucial to effectively discharge their duties. Healthcare workers including nurses should be afforded opportunities for validating their legitimate anxiety and fears related to COVID-19 through periodic screening^6^ of COVID-19-related anxiety^8^ to promote early intervention. However, in Malawi, nurses are not periodically screened for COVID-19-related anxiety.

In Malawi, nurses constitute the bulk of frontline healthcare workers who are fighting the COVID-19 outbreak. However, these nurses amongst other challenges are faced with increased workload and threat of COVID-19, which may aggravate psychological pressure they experience. Some nurses have lamented that one of the hardest things to cope with during the COVID-19 pandemic has been the inconsistencies and constant changes^3^ they experience in their work places. For instance, conflicting and rapidly changing information about personal protective equipment exacerbates healthcare workers’ ongoing fears of exposure and uncertainty about their own safety in the workplace.^6^ Furthermore, protocols for procedures keep on changing rapidly, causing anxiety about whether what they used to do prior to such changes was adequate for themselves and patients.^3^ This shows that nurses and other people who spend much time in thinking about pandemics are at the highest risk of mental illness^5^ including anxiety. Therefore, this study aimed at assessing COVID-19-related anxiety amongst nurses in Malawi. The secondary aim of the study was to determine the reliability and validity of the Coronavirus Anxiety Scale (CAS).

## Methods

### Design

This was a cross-sectional study that collected data from respondents at one point in time to assess COVID-19-related anxiety and functional impairment amongst nurses in Malawi.

### Setting

The study setting was Malawi as a country including all members of the National Organisation of Nurses in Malawi (NONM) working in Christian Health Association of Malawi (CHAM) and government health facilities.

### Study population

The target population were all enrolled nurses, nurse midwife technicians (NMTs) and registered nurses in Malawi. Enrolled nurse and NMTs are the lowest cadre of nurses with a college certificate or diploma in Nursing and Midwifery, whilst registered nurses either have Bachelor of Science in Nursing and Midwifery or Bachelor of Science in Nursing and a University Certificate in Midwifery or University Diploma in Nursing and Midwifery or University Diploma in Nursing and a University Certificate in Midwifery.^10^

### Sample size

The sample size was calculated using the methodology detailed by Jones et al.^11^ This was used to ensure that there were enough cases and non-cases of anxiety for validating CAS. Using estimated specificity = 0.85,^12^ estimated population prevalence of COVID-19-related Anxiety of 0.23^7^ and width of CI = 0.05, a sample size of 255 was calculated. The researcher was most interested in making sure that the test has a high specificity to rule in COVID-19-related anxiety.^11^ However, 320 individuals viewed the survey questionnaire online and a total of 106 respondents completed the questionnaire of which four respondent who were not residing in Malawi were excluded, thus resulting in 102 respondents who participated in this study. This was a convenient sample. The low response rate in this study may have led to sample bias, low power and inaccurate effect size.^13^

### Materials

The data collection instrument for this study included background information, the CAS and the Work and Social Adjustment Scale (WSAS). The instrument was self-administered in English because nurses are expected to understand English by virtue of their training.

#### Background information

Respondents were asked questions related to the background information including age, gender, level of education, employment status, marital status, coronavirus diagnosis, history of anxiety, if they are working with COVID-19 positive patients, if they have a relative or acquaintance with COVID-19 diagnosis,^5^ nursing cadre and workplace.

#### Coronavirus Anxiety Scale

This study used the CAS, which was specifically designed to assess anxiety that is triggered by COVID-19.^12^ The tool is a five-item Likert scale, with each item having five possible responses ranging from 0 (*not at all*) to 4 (*nearly every day over the last 2 weeks*). The CAS has a maximum score of 20 and a minimum score of 0, with an optimum cut-off score of ≥ 9.^12,14^ It is a reliable (Cronbach’s alpha = 0.93) and valid tool for measuring COVID-19-related anxiety (sensitivity = 90%, specificity = 85%, area under the curve [AUC] = 0.94, *p* < 0.001).^12^ The instrument was used to distinguish individuals with dysfunctional anxiety and those without anxiety.

#### Work and Social Adjustment Scale

This study also used an adapted WSAS to measure functional impairment experienced by respondents.^15^ The WSAS is a five-item Likert scale, with each item having nine possible responses ranging from 0 (*not at all impaired*) to 8 (*very severely impaired*). The tool has a maximum score of 40 and a minimum score of 0, with an optimum cut-off score of ≥ 21 for moderately severe or worse psychopathology.^14,15^ Scores of 10–20 suggest significant functional impairment but less severe clinical symptomatology, whilst scores < 10 are associated with subclinical populations. It is a reliable instrument (Cronbach’s alpha ≥ 0.88).^14,15^ The WSAS was used as a gold standard against which the CAS was validated.

### Data collection procedure

Data collection was conducted from August 2020 to September 2020 using a self-administered questionnaire powered by Surveys for Pages and Google Pages. Online links for the study questionnaire were sent to potential respondents through WhatsApp, Facebook, Messenger and email. The questionnaire included information about the study and a question which asked consent from potential respondents before deciding to participate in the study.

### Data analysis

Data were analysed using the Statistical Package for Social Sciences (SPSS) version 25. Descriptive statistics including mean, standard deviation, percentage and frequencies were used to summarise data for background information, the CAS scores and the WSAS scores. Analysis of variance (ANOVA) and independent samples Student’s *t*-tests were used to test for mean difference of respondents’ scores. A receiver operating characteristic (ROC) analysis was used to generate values for sensitivity, specificity, AUC, positive predictive values (PPV), negative predictive values (NPVs) and Youden’s index for the CAS to identify functionally impaired nurses and test whether or not its original cut-off score of ≥ 9 remained an optimal score^12^ for psychiatric screening in the local setting. Finally, Cronbach’s alpha for the CAS and the WSAS were computed to assess their internal consistency locally.

### Ethical considerations

Ethical approval to conduct the study was obtained from the College of Medicine Research and Ethics Committee, University of Malawi (Ethical Clearance number P.08/20/3096, 24 June 2020).

This study received ethical approval and institutional clearance from relevant authorities. The online questionnaire was preceded by an information sheet that explained the nature and benefits of the study to nurses before they were asked to give consent to participate in the study. Respondents’ names did not form part of background information that was collected, thus respecting their privacy and maintaining confidentiality. Respondents were informed that only aggregated data will be analysed and disseminated. Respondents were also informed that their participation in the study was voluntary and that they were free to withdraw at any time if they felt uncomfortable about any aspect during the course of the study. They were further informed that refusing to join the study would not have any effect on their job.

## Results

### Demographic charateristics of respondents

The respondents in this study were drawn from hospitals (56.9%, *n* = 58), nursing colleges (24.5%, *n* = 25), COVID-19 ward (2%, *n* = 2) and non-governmental organisations (NGOs) (16.6%, *n* = 17). They included 71.62% (*n* = 73) female and 28.4% (*n* = 29) male nurses. There were more registered nurses (91.2%, *n* = 93) compared with nurse midwive technichians (8.8%, *n* = 9). The following were educational qualifications of respondents: Bachelor’s degree (52.9%, *n* = 54), Master’s degree (30.4%, *n* = 31), Doctor of Philosophy (5.9%, *n* = 6), Diploma (7.8%, *n* = 8) and Certificate (2.9%, *n* = 3). The employment status of respondents were as follows: full-time (88.2%, *n* = 90), unemployed (9.8%, *n* = 10) and part-time (2%, *n* = 2). Some respondents were married (69.6%, *n* = 71) and others were not (8.8%, *n* = 9). Some respondents (91.2%, *n* = 93) were never diagnosed with COVID-19 whilst others (8.8%, *n* = 9) were diagnosed. More than a quarter of respondents (25.5%, *n* = 26) had a history of anxiety, whilst many (74.5%, *n* = 76) did not have. There were few respondents (13.7%, *n* = 14) who reported that they were working with COVID-19 patients, whilst many (86.3%, *n* = 88) were not. More than one-third of respondents (40.2%, *n* = 41) had a relative with COVID-19, whilst others (59.8%, *n* = 61) did not. The age of respondents ranged from 21 years to 60 years, with a mean age of 36.7 ± 8.9 years.

### Prevalence of COVID-19-related anxiety amongst respondents

This study found that COVID-19-related anxiety was high (25.5%, *n* = 26) amongst nurses in Malawi. There were significant differences in the number of respondents who had COVID-19-related anxiety in relation to workplace (*Χ*^2^ = 8.8, *p* = 0.03) (Table 1). The prevalence of COVID-19-related anxiety was highest amongst respondents who were working in hospitals (36.2%, *n* = 21), with a mean CAS score of 6.7 ± 4.8 (Table 1). The prevalence of COVID-19-related anxiety varied in relation to other demographic characteristics (Table 1). However, this finding was not significant.

**TABLE 1:** Demographic characteristics of respondents associated with COVID-19-related anxiety.

Characteristics	COVID-19-related anxiety						
	Yes†		No‡		Statistic		Mean CAS scores
	*n*	%	*n*	%	*χ*^2^	*p*	
**Age**					0.5	0.5	
≤ 35 years	15	57.7	38	50.0			5.5 ± 4.9
≥ 36 years	11	42.3	38	50.0			4.4 ± 4.7
**Gender**					0.49	0.48	
Female	20	27.4	53	72.6			5.3 ± 5
Male	6	20.7	23	79.3			4.1 ± 4.1
**Education**					1.1	0.9	
Bachelor’s	14	25.9	40	74.1			5.4 ± 4.8
Certificate	1	33.3	2	66.7			8 ± 6
Diploma	3	37.5	5	62.5			6.9 ± 4.7
Master’s	7	22.6	24	77.4			3.8 ± 4.4
Doctorate	1	16.7	5	83.3			2.8 ± 4.6
**Nursing cadre**					1.9	0.17	
NMT	4	44.4	5	56.6			8 ± 4.9
Registered nurse	22	23.7	71	76.3			4.7 ± 4.7
**Employment status**					2.3	0.52	
Full-time	25	27.8	65	72.2			5.1 ± 4.8
Part-time	0	0.0	2	100.0			3 ± 4.2
Unemployed	1	11.1	8	88.9			4.1 ± 4.6
**Workplace**					8.8	0.03*	
Hospital	21	36.2	37	63.8			6.7 ± 4.8
Nursing college	4	16.0	21	84.0			2.8 ± 4.2
COVID-19 ward	0	0.0	2	100.0			1.5 ± 0.7
NGOs	1	5.9	16	94.1			2.5 ± 2.7
**Marital status**					0.3	0.59	
Married	17	23.9	54	76.1			4.8 ± 4.9
Not married	9	29.0	22	71.0			5.3 ± 4.6
**Ever diagnosed with COVID-19**					1.9	0.17	
Yes	4	44.4	5	55.6			8.1 ± 5.6
No	22	23.7	71	76.3			4.6 ± 4.6
**History of anxiety**					0.1	0.7	
Yes	6	23.1	20	76.9			5.8 ± 4.2
No	20	26.3	56	73.7			4.7 ± 4.9
**Working with COVID-19 patients**					0.9	0.35	
Yes	5	35.7	9	64.3			7.4 ± 5.1
No	21	23.9	67	76.1			4.6 ± 4.6
**Having relatives with COVID-19**					0.5	0.47	
Yes	12	29.3	29	70.7			5.7 ± 5
No	14	23.0	47	77.0			4.5 ± 4.5

Note: Data = *n* (%) or mean ± standard deviation.

COVID-19, coronavirus disease 2019; NMT, nurse midwife technician; CAS, Coronavirus Anxiety Scale; NGO, non-governmental organisation.

* , Significance set at ≤ 0.05.

† , *n* = 26, 25.5%;

‡ , *n* = 76, 74.5%.

### Prevalence of functional impairment amongst respondents

This study found that nearly half of the respondents (48.0%, *n* = 49) in this study suffered from functional impairement because of COVID-19. There were variations in number of respondents who suffered functional impairment in relation to demographic characteristics (Table 2). These differences were not significant (*p* > 0.05) except for gender and workplace. The prevalence of functional impairement was significantly higher amongst female respondents (56.2%, *n* = 41) compared with their male counterparts (27.6%, *n* = 8) (*Χ*^2^ = 6.8, *p* = 0.01). There were significant differences in the numbers of respondents who had functional impairement in relation to workplace (*Χ*^2^ = 8.7, *p* = 0.03) (Table 2). The prevalence of functional impairment was highest amongst respondents who were working in hospitals (58.6%, *n* = 34), with mean WSAS score of 20.6 ± 10.4 (Table 2). Furthermore, none of those working in COVID-19 ward (0%, *n* = 0) had functional impairement with the lowest mean WSAS score of 5.5 ± 7.8.

**TABLE 2:** Demographic characteristics of respondents associated with functional impairment.

Characteristics	Severe functional impairment						
	Yes†		No‡		Statistic		Mean WSAS score
	*n*	%	*n*	%	*χ*^2^	*p*	
**Age**					1.0	0.3	
≤ 35 years	28	57.1	25	47.2			20.1 ± 10.4
≥ 36 years	21	42.9	28	52.8			15.7 ± 10.9
**Gender**					6.8	0.01*	
Female	41	56.2	32	43.8			18.8 ± 10.4
Male	8	27.6	21	72.4			15.9 ± 11.7
**Education**					1.7	0.79	
Bachelor’s	28	51.9	26	48.1			19.2 ± 9.9
Certificate	2	66.7	1	33.3			22 ± 7.9
Diploma	3	37.5	5	62.5			18.4 ± 9.6
Master’s	14	45.2	17	54.8			16.3 ± 21.1
Doctorate	2	33.3	4	66.7			13.3 ± 14.6
**Nursing cadre**					0.2	0.64	
NMT	5	56.6	4	44.4			21.9 ± 7.7
Registered nurse	44	47.3	49	52.7			17.6 ± 11
**Employment status**					1.0	0.8	
Full-time	44	48.9	46	51.1			18 ± 10.9
Part-time	1	50.	1	50.0			15 ± 9.9
Unemployed	4	40.0	6	60.0			18.6 ± 11.7
**Workplace**					8.7	0.03*	
Hospital	34	58.6	24	41.4			20.6 ± 10.4
Nursing college	11	44.0	14	56.0			16 ± 11.5
COVID-19 ward	0	0.0	2	100.0			5.5 ± 7.8
NGO	4	23.5	13	76.5			13.6 ± 8.9
**Marital status**					0.7	0.5	
Married	36	50.7	35	49.3			18.7 ± 10.7
Not married	13	41.9	18	58.1			16.4 ± 10.4
**Ever diagnosed with COVID-19**					1.3	0.24	
Yes	6	66.7	43	46.2			21.7 ± 10.8
No	3	33.3	50	53.8			17.6 ± 10.8
**History of anxiety**					1.3	0.7	
Yes	15	57.7	11	42.3			21.2 ± 9.6
No	34	44.7	42	55.3			16.9 ± 11
**Working with COVID-19 patients**					0.5	0.5	
Yes	8	57.1	6	42.9			20.3 ± 12.3
No	41	46.6	47	53.4			17.6 ± 10.5
**Having relatives with COVID-19**					3.0	0.08	
Yes	24	58.5	17	41.5			21 ± 11.2
No	25	41.0	36	59.0			16 ± 10.1

Note: Data = *n* (%) or mean ± standard deviation.

COVID-19, coronavirus disease 2019; NMT, nurse midwife technician; WSAS, Work and Social Adjustment Scale, NGO, non-governmental organisation.

* , Significance set at ≤ 0.05.

† , *n* = 49, 48%;

‡ , *n* = 53, 52%.

The demographic characteristics that were found to have significant differences based on Chi-square test were further analysed using one-way ANOVA to determine if there were any significant differences in respondent scores on the CAS and the WSAS based on workplace. The findings revealed that there were significant differences in the respondents’ mean scores on the CAS (*F* = 3.1, *p* = 0.03) and the WSAS (*F* = 3, *p* = 0.03) based on workplace. Post hoc comparisons using Tukey’s honestly significant difference (HSD) test indicated that the WSAS mean score for the respondents working in the hospital (mean [*M*] = 20.6 ± 10.4) was significantly different (*p* = 0.05) from those working in NGOs (*M* = 13.6 ± 8.9). However, the WSAS mean scores for respondents working in nursing colleges (*M* = 16 ± 11.5) and COVID-19 wards (*M* = 5.5 ± 7.8) did not significantly differ from those working in hospital (*p* > 0.05). Independent samples *t*-tests revealed that female respondents (*M* = 18.8 ± 10.4) had higher WSAS scores than male respondents (*M* = 15.9 ± 11.7) and this result was not significant (*t* = 1.2, *p* = 0.22). A further post hoc comparison using the Tukey’s HSD test showed that the CAS mean scores of the respondents working in the hospital (*M* = 6.7 ± 4.8) were significantly different (*p* = 0.05) from those working in NGOs (*M* = 2.5 ± 2.7). However, the CAS mean score of respondents working in nursing colleges (*M* = 2.8 ± 4.2) and COVID-19 wards (*M* = 1.5 ± 0.7) did not significantly differ from those working in hospital (*p* > 0.05).

### Reliability and validity of the Coronavirus Anxiety Scale

This study revealed that both the CAS (Cronbach’s alpha = 0.9) and the WSAS (Cronbach’s alpha = 0.8) are reliable instruments with good internal consistency for assessing COVID-19-related anxiety and functional impairment in the local setting. A further analysis using the WSAS as a gold standard, the CAS (cut off ≥ 9) was found to be a valid (sensitivity = 73.1%, specificity = 60.5%, NPV = 38.8% and PPV = 86.8%) and accurate (AUC = 0.73) instrument for measuring COVID-19-related anxiety locally. This study found that the optimum cut-off score of the CAS was > 3 (Youden’s index = 0.34), which was lower than the original optimum cut-off score of > 9.

## Discussion

The results of this study are less conclusive considering that the sample size used was small. Nonetheless, COVID-19-related anxiety is one of the mental health problems affecting nurses. The primary aim of this study was to assess COVID-19-related anxiety and functional impairment amongst nurses in Malawi, with the secondary aim being to determine the reliability and validity of the CAS. In this study, more than a quarter of nurses (25.5%, *n* = 26) were found to have COVID-19-related anxiety. This is comparable with a prevalence of COVID-19-related anxiety that was found amongst nurses in China (27.9%).^16^ However, the prevalence of COVID-19-related anxiety found in this study was higher than a pooled prevalence of COVID-19-related anxiety amongst health workers (23.2%) that was reported by a systematic review.^7^ This may be explained by the literature, which asserted that nurses usually experience strong emotional reactions to the COVID-19 virus including anxiety, which impact their work.^2^ In this study, almost half of the nurses (48%, *n* = 49) had self-reported functional impairement related to COVID-19. Mental health problems can significantly reduce the quality of care offered by nurses.^17^ This is corroborated by Pragholapati and colleagues who asserted that COVID-19-related anxiety can interfere with performance of nurses in their duties and reduce their self-efficacy levels.^9^

This study suggests that many nurses who work in hospitals experience high levels of anxiety (36.2%, *n* = 21, Mean CAS score = 6.7 ± 4.8) and functional impairment (58.6%, *n* = 34, mean WSAS score = 20.6 ± 10.4). As frontline healthcare workers, nurses may be vulnerable to negative mental health effects from COVID-19. The nature of care itself and new ways of working are potentially highly stressful for nurses who are being overwhelmed with increased workload and demands to accommodate new protocols.^2^ Literature indicated that it is necessary to pay attention to the psychological issues of nurses during and after caring for patients with COVID-19.^17^ Supporting nurses practically and psychologically helps in preserving their health especially when work-related stress levels are too high.^2^

Hospitals should provide sufficient support to nurses, including personal protective equipment, psychological screening for nurses and psychological support.^17^ This is crucial because nurses may not be able to recognise and deal with their own mental health problems whilst they provide care to COVID-19 patients. This is supported by a body of literature, which suggested that those injured by stress may be the last to recognise it and individuals often do not prioritise taking good care of themselves.^2^ It is necessary that nurses with symptoms of anxiety seek help from psychotherapists to evaluate them and help them deal with potential stress.^18^ This is helpful because nurses must be able to look after themselves if they are going to properly look after others.^2^ However, nurses are generally trained to look after others and not self so that in many instances they need others including colleagues, friends and managers to remind them to think of themselves.^2^

Reliable and valid instruments are needed for nurses to detect and validate their COVID-19-related anxiety. As a result of concern about the variation of performance of screening instruments in different populations and settings,^19^ it was necessary to measure the validity of the CAS in the local setting. This study showed that the CAS was able to distinguish nurses with anxiety from those without anxiety (sensitivity = 73.1%, specificity = 60.5%, AUC = 0.73, cut-off ≥ 9) in the local setting. The results support the CAS as an effective, reliable (Cronbach’s alpha = 0.9) and valid instrument for clinical research and practice.^5,12,14^

This study had some limitations because it was conducted online and those without Internet access or without adequate Internet bundles could not be involved in the study. These results are biased towards registered nurses who managed to respond to online questionnaire because they have a relatively better income as compared with NMTs. Future research should consider using an alternative means of getting data such as face-to-face data collection methods to ensure adequate representation of all cadres of nurses. Furthermore, the results of this study heavily relied on respondents’ self-reports, which may have yielded recall bias because nurses were asked to recall events that happened two weeks or more ago. There was no common attention check item (instructed response item) embedded within the online survey. Consequently, the validity of the CAS might have been affected by careless responses.

## Conclusion

This study suggested that the prevalence of COVID-19-related anxiety is high amongst nurses in Malawi. Most nurses experience functional impairment because of COVID-19. Furthermore, this study has confirmed that the CAS is a valid instrument which is effective in detecting COVID-19-related anxiety amongst nurses. The CAS could be a suitable instrument for assessing COVID-19-related anxiety amongst nurses.

### Implications for practice

Considering that prevalence of COVID-19-related anxiety is high amongst nurses, it is necessary to screen nurses for the COVID-19-related anxiety and functional impairment and provide them effective psychosocial interventions. Mental health interventions targeting nurses should include preparedness to reduce the effects of COVID-19 on their mental health and well-being. Nurses need moral, psychological and material support from their employers and colleagues. It is important that policymakers allocate adequate funding to mental health services targeting nurses during pandemics. There is a need to conduct future research on mental health interventions that might be used to assist nurses with COVID-19-related anxiety and functional impairment.
